# Intravenous Continuous Infusion vs. Oral Immediate-release Diltiazem for Acute Heart Rate Control

**DOI:** 10.5811/westjem.2017.10.33832

**Published:** 2018-02-22

**Authors:** Kimberly N. Means, Amanda E. Gentry, Tammy T. Nguyen

**Affiliations:** * Virginia Commonwealth University Medical Center, Department of Pharmacy, Richmond, Virginia; † Virginia Commonwealth University, Department of Biostatistics, Richmond, Virginia; ‡ Virginia Commonwealth University Medical Center, Department of Emergency Medicine, Richmond, Virginia

## Abstract

**Introduction:**

Atrial fibrillation (AF) is a common diagnosis of patients presenting to the emergency department (ED). Intravenous (IV) diltiazem bolus is often the initial drug of choice for acute management of AF with rapid ventricular response (RVR). The route of diltiazem after the initial IV loading dose may influence the disposition of the patient from the ED. However, no studies exist comparing oral (PO) immediate release and IV continuous infusion diltiazem in the emergency setting. The objective of this study was to compare the incidence of treatment failure, defined as a heart rate (HR) of >110 beats/min at four hours or conversion to another agent, between PO immediate release and IV continuous infusion diltiazem after an initial IV diltiazem loading dose in patients in AF with RVR.

**Methods:**

This was a single-center, observational, retrospective study conducted at a tertiary academic medical center. The study population included patients ≥18 years old who presented to the ED in AF with a HR > 110 beats/min and received an initial IV diltiazem loading dose. We used multivariate logistic regression to assess the association between routes of administration and treatment failure.

**Results:**

A total of 111 patients were included in this study. Twenty-seven percent (11/41) of the patients in the PO immediate-release group had treatment failure compared to 46% (32/70) in the IV continuous-infusion group. The unadjusted odds ratio (OR) of treatment failure with PO was less than IV at 0.4 (95% confidence interval [CI] [0.18, 0.99], p = 0.046). When we performed a multivariate analysis adjusted for race and initial HR, PO was still less likely to be associated with treatment failure than IV with an OR of 0.4 (95% CI [0.15, 0.94], p = 0.041). The median dose of PO diltiazem and IV continuous infusion diltiazem at four hours was 30 mg and 10 mg/h, respectively.

**Conclusion:**

After a loading dose of IV diltiazem, PO immediate-release diltiazem was associated with a lower rate of treatment failure at four hours than IV continuous infusion in patients with AF with RVR.

## INTRODUCTION

Atrial fibrillation (AF), a supraventricular tachyarrhythmia, is the primary diagnosis for over 467,000 hospitalizations each year.^1^ Historically, there have been two approaches to managing AF in the emergency department (ED): rate control and rhythm control.

The AFFIRM trial compared rate and rhythm control in 4,060 chronic AF patients. It found no difference in overall mortality, but there were fewer hospitalizations with rate control compared to rhythm.^2^ The subsequent RACE II trial established that lenient heart rate (HR) control (HR <110 beats/min) was as effective as strict control (HR <80 beats/min) in preventing cardiovascular events and required fewer outpatient visits to achieve the goal HR.^3^ A number of medications are used for rate control including beta blockers and non-dihydropyridine calcium channel blockers.^1^

Diltiazem, a non-dihydropyridine calcium channel blocker, is a common initial choice in the management of AF with rapid ventricular response (RVR) due to its ability to be given as an intravenous (IV) push, continuous infusion, and oral (PO) immediate-release or extended-release tablet. In the ED a loading dose (LD) of IV diltiazem is usually administered followed by PO immediate-release tablet or IV continuous infusion. Both options allow for dose titration in the short term before converting to a longer-acting PO formulation for discharge. The PO immediate-release diltiazem tablet has an onset of action of 30–60 minutes and is dosed every six hours.^4^ IV continuous infusion diltiazem has a rapid onset of action and is titrated every 15–30 minutes.

The route of diltiazem after the initial IV LD can influence the disposition of the patient from the ED, the level of care needed, and hospital length of stay (LOS). Patients who receive only the PO immediate-release diltiazem absorb a therapeutic dose quickly and can generally be discharged or admitted to a general medicine floor, but cannot be titrated more frequently than every six hours. Patients who received the IV continuous infusion must have their dose frequently titrated by nursing and often require stepdown care. No studies exist comparing the efficacy of PO immediate-release and IV continuous-infusion diltiazem in the emergent management of AF with RVR. The objective of this study was to compare the incidence of treatment failure at four hours between PO immediate-release and IV continuous-infusion diltiazem after an IV LD.

## METHODS

### Study Design

This was a retrospective, observational, medical record review conducted with data from Virginia Commonwealth University Health, a tertiary medical center ED that treats over 95,000 patients annually. We retrospectively identified cases of ED diltiazem use from July 1, 2014, to July 1, 2015, from electronic medical records. Inclusion criteria included the following: patients ≥ 18 years old who presented to the ED in AF, with a HR > than 110 beats/min, who received an initial diltiazem IV LD and then subsequently PO immediate-release or IV continuous-infusion diltiazem. Of note, no ED AF protocol existed at the time of the study. Diltiazem dose and route selection were at the discretion of the ED provider. We excluded patients if they were pregnant or a prisoner. We also excluded patients if they had received electrical cardioversion or other rate control or antiarrhythmic medication in the prehospital or ED setting before being administered an IV LD of diltiazem. This study was approved by the institutional review board.

**Population Health Research Capsule**

What do we already know about this issue?

Diltiazem is often used in the acute, emergent management of atrial fibrillation. No studies exist comparing oral (PO) immediate release and intravenous (IV) continuous infusion diltiazem.

What was the research question?

Compare the incidence of treatment failure at four hours between PO immediate release and IV continuous infusion diltiazem.

What was the major finding of the study?

PO immediate-release diltiazem was associated with a lower rate of treatment failure at four hours than IV continuous infusion.

How does this improve population health?

If PO immediate-release diltiazem is associated with less treatment failure, it may permit the disposition of emergency department patients to a less resource-intensive setting.

### Study Protocol

We collected and managed study data using REDCap^®^ electronic data capture tools.^5^ Baseline demographic information recorded included the patient’s age, sex, race, and weight. Diltiazem dosing characteristics at baseline and four hours and the use of adjunctive medication for HR or rhythm control at four hours were collected. Clinical outcomes recorded included HR and blood pressure (BP) at baseline and four hours, ED disposition, and hospital LOS.

Two of the study’s investigators abstracted all available data independently. Both were involved in the study design and used a standardized data collection form in REDCap^®^ that included study definitions to ensure consistency between the investigators. Investigators were not blinded to the study outcome. Any discrepancies between abstractors resulted in a collaborative review of the chart by both investigators until discrepancies were resolved. As a result, interrater reliability was not determined.

### Measures

The primary endpoint of the study was the percentage of patients with treatment failure at 4 ± 1 hour after initiation of PO immediate-release diltiazem or continuous IV diltiazem infusion. Treatment failure was defined as HR of > 110 beats/min at 4 ± 1 hour, a switch in therapy from PO immediate-release diltiazem to IV continuous infusion diltiazem, the requirement of an additional IV diltiazem bolus within four hours from the start of PO or IV continuous infusion, or addition/switch of therapy to another rate control or antiarrhythmic agent within four hours. A clinical endpoint of 4 ± 1 hour was selected to give time for both the PO and the IV diltiazem to have therapeutic effect. It was also concluded that this was a reasonable amount of time for the ED provider to determine disposition. We made the decision not to include time points extending beyond four hours due to the increased number of confounding factors, including the conversion to PO β-blockers or extended-release PO diltiazem.

Patient characteristics collected included age, weight, race, sex, initial HR and BP, and initial diltiazem LD. We assessed the safety endpoint of clinically significant hypotension by recording the indication for diltiazem discontinuation and the need for vasopressors administration for hemodynamic support.

### Sample Sizes and Data Analysis

No power calculation was done due to the study’s exploratory nature. We based dates for study inclusion on when diltiazem PO immediate-release tablets became readily available in the ED medication-dispensing unit. If included patients presented to the ED multiple times during the study period, only the most recent encounter was considered.

We analyzed data using Excel, R 3.2.2, and JMP 11.0.0 (copyright 2013 SAS Institute, Cary, NC). Nominal variables were evaluated with Χ^2^ or Fisher’s exact test, and we compared continuous variables using Student’s t-test. We used univariate logistic regression to identify those characteristics associated with treatment failure and therefore eligible for inclusion in a final, multivariable model. Per the modeling strategy presented by Hosmer et al., a liberal p-value of 0.15 was used to identify these potential confounders.^6^ We used multivariable logistic regression to control for these confounding characteristics while modeling the association between dosing route and treatment failure at four hours. An a priori α level of ≤0.05 was used to determine statistical significance.

## RESULTS

**Figure 1:**
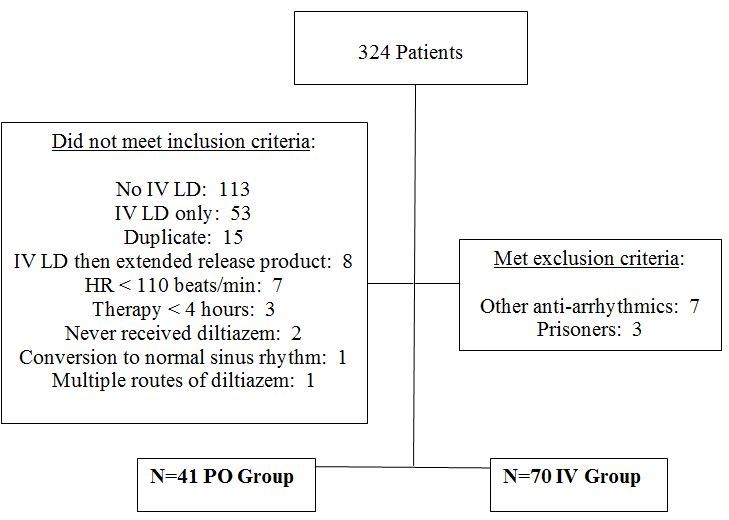
Inclusion/exclusion criteria and treatment assignment. *PO*, oral; *IV*, intravenous; *LD*, loading dose; *HR*, heart rate.

We reviewed 324 patients for study inclusion and excluded 213 (Figure 1). The most common reasons for exclusion were the lack of an IV diltiazem LD, the administration of an IV diltiazem LD only, and duplicate encounters. Complete data were available for 111 patients, 41 in the PO immediate-release diltiazem and 70 in the IV continuous-infusion diltiazem groups. Study population demographics are reported in Table 1. The overall mean age was 62 years, with 52% male gender and a mean weight of 93 kg. When PO immediate-release diltiazem and the IV continuous-infusion diltiazem groups were compared, the only baseline characteristic that was significantly different between the two was the mean initial HR. The PO group had an initial HR of 131 +/− 19 beats/min compared to the IV group which had an initial HR of 145 +/− 18 beats/min (P=0.002).

For the primary endpoint of treatment failure at four hours, 27% of patients (11/41) in the PO immediate-release diltiazem group met criteria compared to 46% of patients (32/70) in the IV group, a difference of 19% (p=0.049). The unadjusted odds ratio (OR) of treatment failure with PO when compared to IV was 0.4 (95% confidence interval [CI] [0.18, 0.99], p=0.046) (Table 2). We performed a multivariate analysis adjusting for initial HR and race. Mean initial HR was included due to the statistically significant difference in baseline characteristics. We included race from the univariate logistic regression models because the p-value was below the 0.15 threshold (Table 2). Although ED disposition was significantly different between the groups, we did not include it in the multivariable logistic regression since it was a secondary outcome of interest and not a potential confounder. In the multivariate model, the adjusted odds of treatment failure at four hours with PO compared to IV remained statistically significant at 0.4 (95% CI [0.15, 0.94], p=0.041). A HR of >110 at four hours accounted for 25 of 32 treatment failures in the IV group compared to nine of 11 in the PO immediate-release group.

Fifty-three percent of patients in the PO immediate-release diltiazem group received an initial dose of 30 mg and 41% received 60 mg. The median dose of IV continuous-infusion diltiazem at four hours was 10 mg/h (range 2.5 mg/h to 20 mg/h). Patient disposition from the ED can be seen in Table 1, with a statistically significant difference in the disposition between PO and IV (P<0.0001). The odds of disposition to a general floor were 6.1 times higher (95% CI [2.47 – 15.92], P < 0.0001) with PO compared to IV. Patients in the PO group were less likely than IV to be admitted to the stepdown or intensive care unit (ICU) with an OR of 0.3 (95% CI [0.10 – 0.80], P = 0.0112), and 0.2 (95% CI [0.02 – 0.69], p-value 0.0051), respectively. We found no statistically significant difference in discharge to home with an OR 1.4 (95% CI [0.26 – 6.96], P = 0.7234) due to the small sample size. The mean and median LOS was 4.7 days and three days, respectively, in the PO group and nine days and five days, respectively, in the IV group.

From a safety standpoint, no patients required vasopressors for BP support or had their diltiazem therapy discontinued for hypotension. Diltiazem was stopped for only two indications in both the PO and IV group-change in agent and lack of indication (i.e., the patient’s AF had resolved). In one case, the discontinuation reason was unknown.

## DISCUSSION

In the emergent setting, diltiazem has been shown to be superior to digoxin, metoprolol, and amiodarone in the initial management of AF and flutter.^1^,^7^–^10^ IV diltiazem has often been considered superior to PO in the management of AF due its 100% bioavailability and titratability. However, PO immediate-release diltiazem confers many benefits over IV continuous infusion including a fast onset of action, minimal titration requirement, decreased nursing resources, and the ability to disposition to a general floor or possibly discharge home. A comparison of PO immediate-release and IV continuous-infusion diltiazem in the emergent clinical setting had never been performed.

In our study, we found that PO immediate-release diltiazem resulted in a 0.4 (95% CI 0.15–0.94) OR of treatment failure when compared to IV continuous infusion. In other words, PO immediate-release diltiazem resulted in an odds of heart rate control 2.6 times greater than IV continuous infusion at four hours. This is a surprising result given the higher bioavailability of the IV route compared to the oral formulation. A possible reason for this difference in treatment failure may be that IV continuous infusion was sub-optimally titrated. In our sample, the median hourly dose of the IV continuous infusion at four hours was only 10 mg/h, well below the maximum dose of 15 mg/h. Slow titration to sub-maximal doses may have resulted in suboptimal diltiazem plasma concentrations in comparison with patients who were given immediate-release PO diltiazem. In theory, PO dosing may have achieved a higher plasma concentration as a result of the entire diltiazem dose being given at once. Therefore, our results may not reflect the comparison of two treatment regimens at optimal dosing capacity, but rather the real-world practice in which medication titration is not always optimized.

PO diltiazem was associated with statistically significant higher odds of being admitted to the general floor and lower odds of being admitted to stepdown or the ICU. Patients who received PO also had a two-day shorter median LOS compared to IV. While the differences in these two parameters cannot be ascertained in a definitive manner due to the retrospective nature of the study, it is possible that the extended time needed to transition patients from IV to PO diltiazem before discharge may have played a contributing factor. Patient disposition and decreased LOS represent a possible area of healthcare cost savings that should be investigated in future prospective studies.

Providers may choose IV continuous-infusion diltiazem if they want to titrate to lower doses in patients with borderline hemodynamic stability. In our study, however, clinically significant hypotension (defined as hypotension requiring discontinuation of the therapy and/or vasopressors) did not occur in the PO or IV group. Overall, our findings call in to question the primacy of IV continuous-infusion diltiazem for AF. PO diltiazem was associated with a lower rate of treatment failure and higher rate of heart control than IV continuous infusion and with similar safety. Importantly, these findings are the result of a retrospective study with limited sample size and therefore must be confirmed in a larger, prospective, randomized controlled trial.

## LIMITATIONS

This study has several limitations. Its retrospective nature limited sample size and abstraction. Incomplete documentation prevented characterization of the severity of the patient’s symptoms, past medical history of AF, and home medications. In addition, identifying the total amount of diltiazem received via the continuous infusion route to allow for summative dose comparisons against oral was not possible due to inconsistent documentation of IV titrations. The statistically significant difference between groups in baseline HR suggests a potential selection bias against IV continuous-infusion diltiazem as providers may have selected this route of administration for more acutely ill patients and reserved PO diltiazem for milder cases. While we accounted for a select number of patient-specific factors in our logistic regression model, the potential for additional, unmeasured confounders still exist, which could mean the study showed only association, not causation. Our primary endpoint measured treatment failure at 4 ± 1 hour to give time for both the PO and the IV diltiazem to have therapeutic effect. A majority of patients failed due to HR > 110; our study may have excluded other time points where HR control was achieved. Lastly, the small sample size and low power resulted in large CI for the odds ratios.

## CONCLUSION

After a loading dose of IV diltiazem, PO immediate-release diltiazem was associated with a lower rate of treatment failure at four hours when compared to IV continuous infusion in patients with atrial fibrillation with rapid ventricular response.

## Figures and Tables

**Figure 2:**
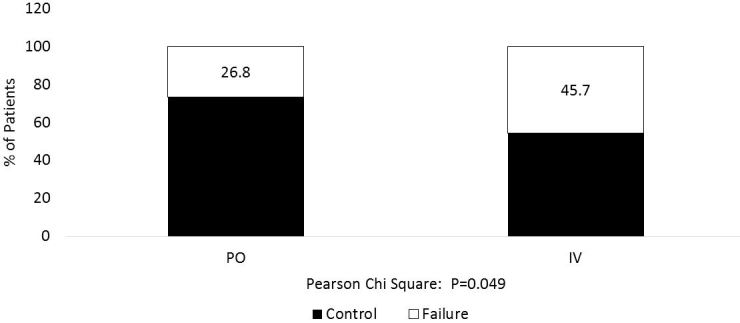
Percentage of patients with treatment failure at four hours. PO, oral; IV, intravenous

**Table 1:** Charachteristic of the sample.

Variable	Overall summary N=111 (SD)	PO group N=41 (SD)	IV group N=70 (SD)	P-value (for t-test or χ2)
Age	62 (13.8)	62 (13.6)	61 (14.1)	0.698
Sex (Male)	58 (52%)	20 (49%)	38 (54%)	0.575
Race (Caucasian)	49 (44%)	17 (41%)	32 (46%)	0.663
Weight (kg)	93 (28.5)	89 (24.6)	95 (30.6)	0.316
Mean initial HR (beats/min)	140 (19.7)	131 (18.6)	145 (18.4)	0.002
Mean initial SBP (mmHg)	133 (26.2)	136 (23.5)	131 (27.7)	0.359
Mean initial DBP (mmHg)	87 (20.9)	91 (17.6)	85 (22.4)	0.109
Mean initial diltiazem dose (mg/kg)	0.23 (0.124)	0.22 (0.108)	0.24 (0.133)	0.579
ED Disposition				<0.0001
Discharge	9 (8%)	4 (10%)	5 (7%)	
General floor	46 (41%)	28 (68%)	18 (26%)	
Stepdown	36 (32%)	7 (17%)	29 (41%)	
Intensive care unit	20 (18%)	2 (5%)	18 (26%)	

*PO*, oral; *IV*; intravenous; *Kg*; kilogram; *HR*, heart rate; *SBP*, systolic blood pressure; *DBP*, diastolic blood pressure; *mmHg*, millimeter of mercury; *mg*, milligram; *SD*, standard deviation.

**Table 2:** Characteristics associated with treatment failure at four hours when comparing use of oral immediate-release diltiazem vs. intravenous continuous infusion.

Variable	Unadjusted odds ratio (95% CI)	P-value	Adjusted odds ratio (95% CI)	P-value
PO diltiazem	0.4 (0.18 – 0.99)	0.046	0.4 (0.15 – 0.94)	0.041
Age (per 5 year decrease)	1.1 (0.92 – 1.22)	0.436		
Male gender	1.3 (0.58 – 2.70)	0.567		
Non-Caucasian race (Reference level: Caucasian)	1.9 (0.86 – 4.17)	0.116	2.0 (0.89 – 4.49)	0.1
Weight (per each 5 kg decrease)	1.0 (0.94 – 1.08)	0.781		
Initial IV loading dose (per 0.1 mg/kg increase)	1.2 (0.89 – 1.66)	0.229		
Initial HR (per 5 beat/min increase)	1.0 (0.92 – 1.12)	0.805	1.0 (0.92 – 1.15)	0.688

*PO*, oral; *IV*, intravenous; *HR*, heart rate; *kg*, kilogram; *mg*, milligram; *CI*, confidence interval.
